# Effective passivation of Ag nanowire-based flexible transparent conducting electrode by TiO_2_ nanoshell

**DOI:** 10.1186/s40580-016-0080-z

**Published:** 2016-08-20

**Authors:** Dong Geon Lee, Dongjun Lee, Jin Sun Yoo, Sangwook Lee, Hyun Suk Jung

**Affiliations:** 1 School of Advanced Materials Science and Engineering, Sungkyunkwan University, Suwon, 16419 South Korea; 2 School of Materials Science and Engineering, Kyungpook National University, Daegu, 41566 South Korea

**Keywords:** TiO2, Sheet Resistance, Atomic Layer Deposition, Silver Nanowires, Mesoporous TiO2

## Abstract

Silver nanowire-based flexible transparent electrodes have critical problem, in spite of their excellent electrical and optical properties, that the electrical conductance and transparency degrade within several days in air because of oxidation of silver. To prevent the degradation of the silver nanowire, we encapsulated Ag-NWs with thin TiO_2_ barrier. Bar-coated silver nanowires on flexible polymer substrate were laminated at 120 °C, followed by atomic layer deposition of TiO_2_ nanoshell. With 20 nm of TiO_2_ nanoshells on silver nanowires, the transparent electrode keeps its electrical and optical properties over 2 months. Moreover, the TiO_2_-encapsulated silver nanowire-based transparent electrodes exhibit excellent bending durability.

## Background

Worldwide demand on wearable or flexible optoelectronic devices has promoted research on various flexible display and power source devices, such as flexible light emitting diodes and flexible solar cells [1, 2]. For actual flexible optoelectronic devices, it is essential to develop flexible transparent conducting electrodes (TCE) which exhibit sufficient transparency in visible light and electrical conductance under bending. Most of the existing flat, rigid optoelectronic devices have been built on transparent conducting oxide (TCO)-based TCEs, representatively tin-doped indium oxide (Sn:In_2_O_3_, ITO) which can be designed to have a high transmittance >85 % and low sheet resistance <8 Ω/sq due to its large band gap energy (~4 eV) and low electrical resistivity (~10^−4^ Ω cm) [3]. However, brittleness of oxide ceramics is not suitable for application to the flexible devices as flexible TCE.

As alternative to the rigid TCOs, various flexible TCEs have been studied using carbon materials such as carbon nanotubes and graphene, conducting polymers such as poly(3,4-ethylenedioxythiophene) polystyrene sulfonate (PEDOT:PSS), or metal nanowires (NWs) such as Ag-NWs and Cu-NWs [4, 5]. Many of the recently developed flexible TCEs have several problems such as lacking junctions between the conducting materials or low stability under ambient circumstance [6], where as they have comparable transmittances and sheet resistances with TCOs. Among various TCEs, Ag-NWs have been intensively studied due to its excellent conductivity and flexibility [7, 8]. Ag-NWs-based TCEs usually have random networks which can be achieved via inexpensive solution processes such as bar coating, spin coating, spray coating and drop casting [9–11]. In this random network, conductivity can be increased by enhancing contacts at the junctions of Ag-NWs, via laminating for instance [12]. However, Ag-NWs have the critical stability issue that is oxidation of Ag to Ag_2_O_3_ in air. Such an oxidation significantly affects long-term sheet resistance of the TCE [13]. For this reason, there have been various attempts to prevent oxidation of Ag-NWs. One of the most effective methods for anti-oxidation is formation of passivation layers on Ag-NWs, such as graphene/Ag or ZnO/Ag [13, 14].

In this study, we report an effective passivation of solution processed Ag-NWs-based flexible TCEs, by formation of stable TiO_2_ nanoshells on the surface of the TCEs, as a barrier to oxidation of the Ag-NWs. To fabricate control TCEs, wet-chemically synthesized Ag-NWs were bar-coated on a polymer substrate followed by laminating. We investigated sheet resistance and transmittance of the control TCEs with varying the number of NW coating, to obtain a high performance TCE. Some of the identically processed TCEs were subjected to deposition of the TiO_2_ nanoshell via atomic layer deposition (ALD). Oxidation of the Ag-NWs with and without the TiO_2_ nanoshell was compared after storage in air during 2 months. Effect of the TiO_2_ barrier on the sheet resistance and the transmittance was also studied with varying the thickness of the TiO_2_ nanoshell. Finally, we tested bending durability of the Ag-NWs-based TCEs, and performance of a solid-state dye-sensitized solar cell (ssDSSC) which is fabricated on the Ag-NWs-based TCEs, up to 100 cycles of bending test with 30 mm of bending radius.

## Results and discussion

**Fig. 1:**
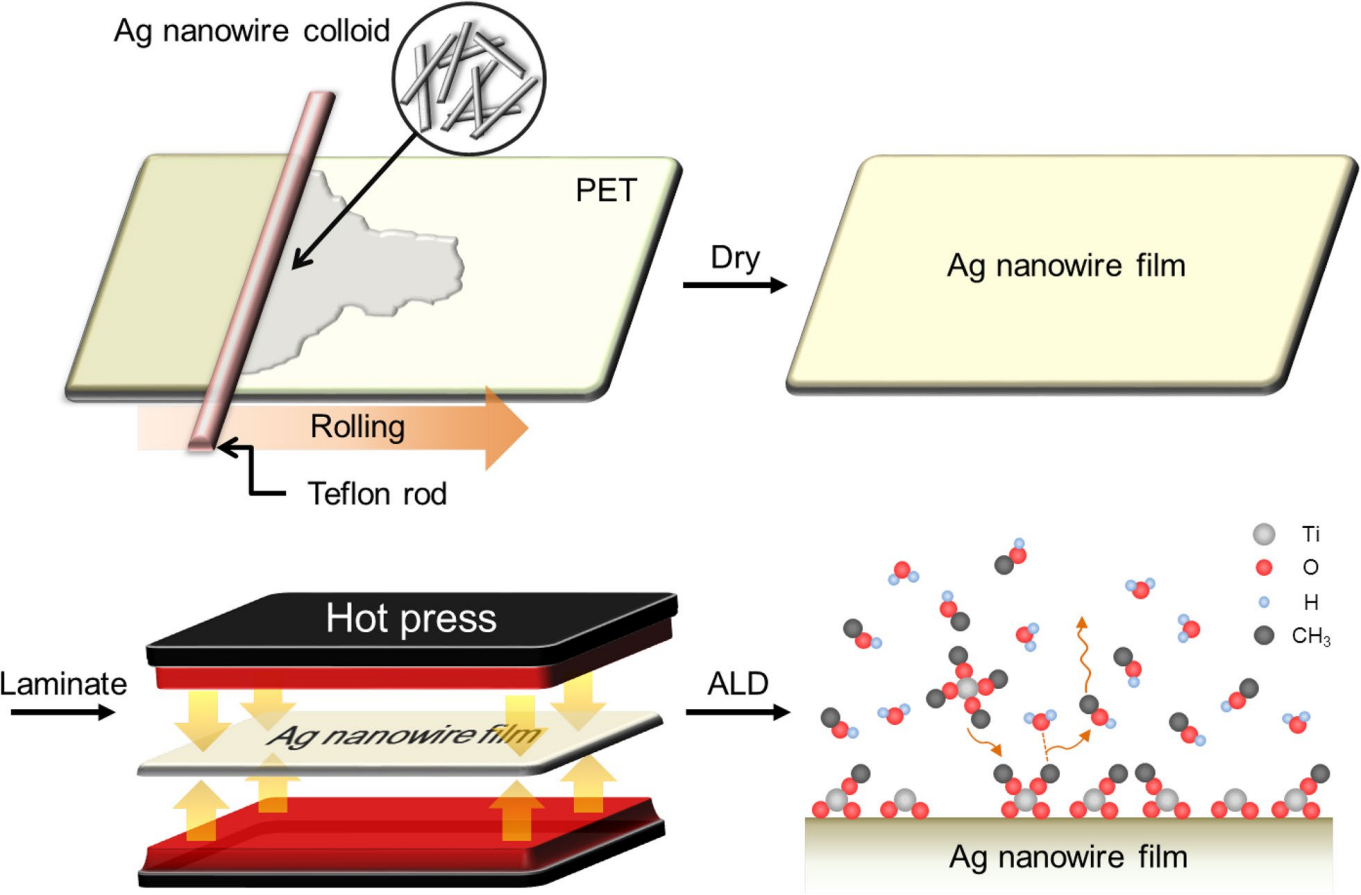
A schematic diagram of the fabrication processes. Ag-NW solution is bar-coated on a PET substrate and dried under an IR lamp, repeatedly. After several cycles of the bar-coating, the Ag-NW/PET is laminated, followed by ALD for the deposition of TiO_2_ nanoshell

Figure 1 shows the process how the Ag-NW TCEs were prepared. Ag-NWs were synthesized via polyol method (details in experimental part). The synthesized Ag-NW solution was uniformly coated onto a flexible polyethylene terephthalate (PET) substrate using a Teflon bar, and dried under the IR lamp. The bar coating method is very simple and controllable, which facilitates scalable roll-to-roll process [10, 12]. Using this method Ag-NWs film is directly formed onto a PET substrate at room temperature. We repeated this process to increase the Ag-NW film density until the network overcomes the percolation threshold, which would result in a more uniform distribution and lower sheet resistance in a film. As-coated Ag-NW film was laminated at 120 °C to ensure interconnections between crossing Ag-NWs and to enhance adhesion between the Ag-NWs and PET substrate. For the last, the Ag-NW TCE was subjected to ALD for deposition of TiO_2_ thin layer to passivate the Ag-NWs from oxidation in air.

**Fig. 2:**
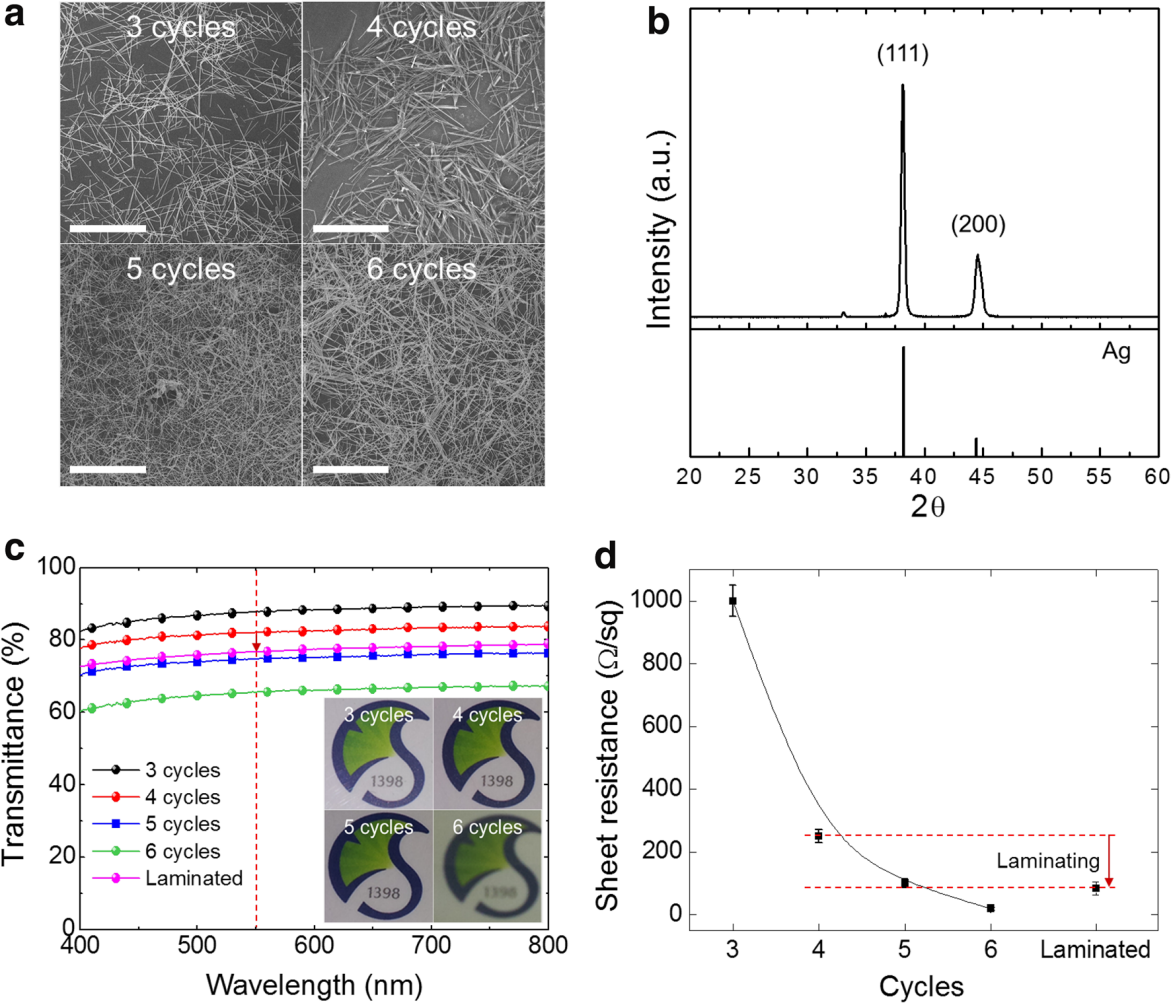
Characterization of the Ag-NW TCEs before TiO_2_ nanoshell coating. **a** SEM images (*scale bar* 20 μm), **b** X-ray diffraction pattern, **c** transmittance (inset: optical images), and **d** sheet resistance of Ag-NW TCEs as a function of the Ag-NW coating cycles. Laminated Ag-NW TCE (4 cycles) is shown in **c** and **d** for comparison

Figure 2a shows top-view scanning electron microscopic (SEM) images of the Ag-NWs on PET, before laminating, with different NWs coating cycles. Density of NWs film increases as the number of coating cycle increases. The prepared film is comprised of the face-centered cubic structured Ag, as identified by the X-ray diffraction pattern shown in Fig. 2b. The Ag-NW TCEs without TiO_2_ deposition were prepared with different coating cycles. Figure 2c, d show the UV–vis transmittance and sheet resistance of the Ag-NW film with varying the coating cycles. The transmittance of the TCE decreases gradually, from 87.68 to 65.51 % at 550 nm of wavelength, with repeating the NW coating. Six cycles of coating results in a translucent film as shown in the inset of Fig. 2c. On the other hand, Fig. 2d shows that the sheet resistance steeply decreases from three cycles to four cycles, and then gently decreases till six cycles. The sheet resistance is 1000, 250, 100 and 20 Ω/sq for the Ag-NW TCEs coated with three, four, five, and six cycles, respectively. All the laminated TCEs, under 200 kg/cm^2^ at 120 °C, resulted in simultaneous decrease of transmittance and sheet resistance. Therefore, to retain reasonable transmittance and sheet resistance, four times coated TCE (laminated) was selected for the further studies, and the data are shown in Fig. 2c, d; 76.69 % of transmittance at 550 nm wavelength, and 84 Ω/sq of sheet resistance.

**Fig. 3:**
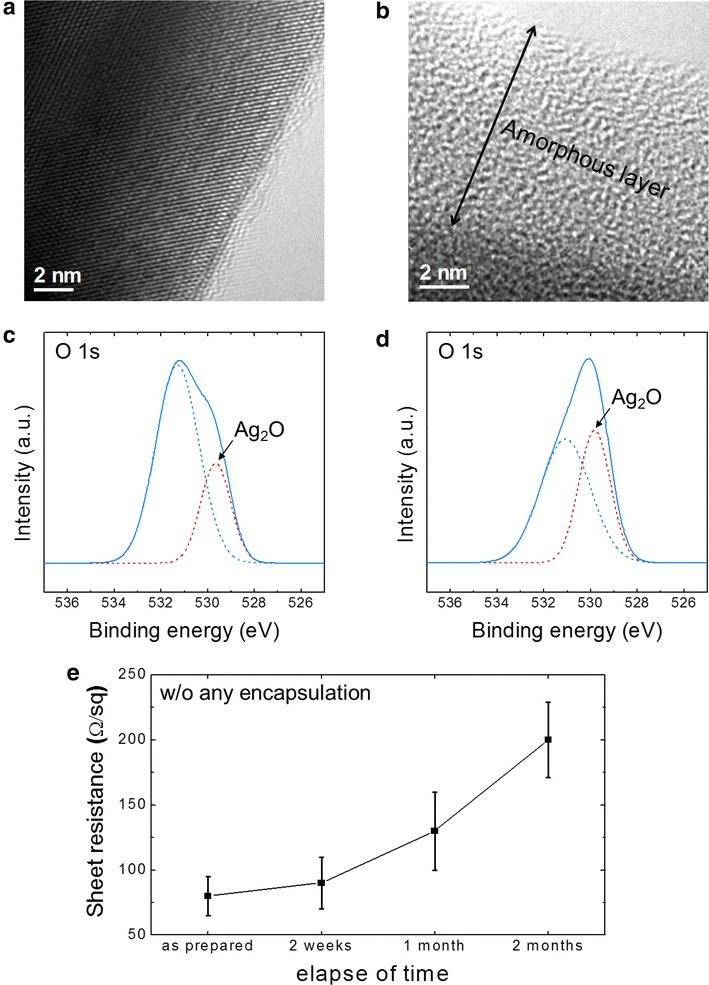
TEM images of an Ag-NWs collected from the laminated TCE; **a** as-fabricated and **b** stored 2 months in air. XPS spectra of an Ag-NW TCE; **c** as-fabricated and **d** stored 2 months in air. **e** Sheet resistance of an Ag-NW TCE as a function of elapse time

In order to confirm the oxidation of Ag-NW when it is open to ambient air without any passivation, we compare the surface crystal structure and chemical states of an as-prepared Ag-NW and those of aged Ag-NW during 2 months in air, using transmission electron microscopy (TEM) and X-ray photoemission spectroscopy (XPS), as shown in Fig. 3a, b and c, d. TEM images show that well-crystallized surface (Fig. 3a) of the as-prepared Ag-NW has been transformed to amorphous phase, evidenced by absence of the lattice fringes in Fig. 3b. Further, XPS spectra show that O 1s peak at 529.8 eV which is corresponding to Ag_2_O increases significantly after the aging in air. The oxidation of Ag-NW affects severely the electrical property of the Ag-NW TCE. Figure 3e shows increasing sheet resistance of the Ag-NW TCE, accelerating with aging time in ambient air, by ~four times within only 2 months. Such a severe degradation nature is not suitable for use of the TCE as an electrode of any real device, because a TCE is required to have a low sheet resistance (<100 Ω/sq). Moreover, change of sheet resistance with time cannot give reliability of the device [15].

**Fig. 4:**
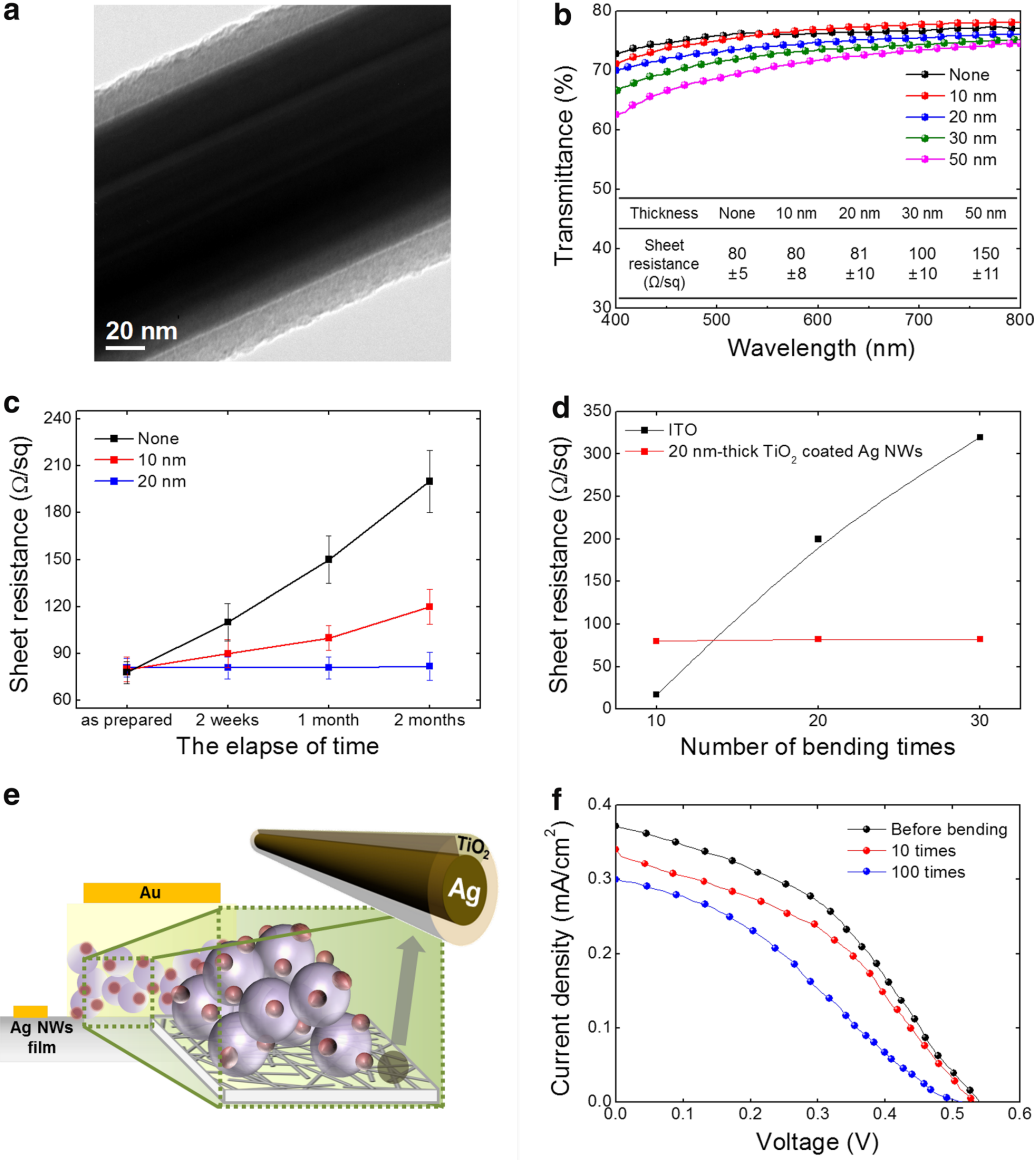
Characterizations of Ag-NW TCEs coated with TiO_2_ nanoshell. **a** TEM image of a single Ag-NW with TiO_2_ nanoshell. **b** Transmittance versus sheet resistance depending on the TiO_2_ nanoshell thickness. **c** Sheet resistance as a function of storage time in air. **d** Sheet resistance of ITO and the 20 nm-thick TiO_2_-coated Ag-NW TCE with mechanical bending (30 mm of bending radius). **e** Schematic diagram of a flexible ssDSSC using the TiO_2_-coated Ag-NW TCE. (*purple sphere* mesoporous TiO_2_ particle, *plum sphere* dye) **f** J-V *curves* of the flexible ssDSSC after 0, 10 and 100 times of bending (30 mm of bending radius)

To prevent the Ag oxidation, thin TiO_2_ barriers were coated on the laminated Ag-NW TCEs using ALD. The thickness of the TiO_2_ passivation layer is varied as 10, 20, 30 and 50 nm. Figure 4a shows a TEM image of the 20 nm-thick TiO_2_ coated Ag-NW. The dark core, and the distinct bright and uniform shell confirms successful formation of the TiO_2_ shell. Figure 4b shows transmittance and sheet resistance (inset table) of the TiO_2_-coated Ag-NW TCE, with various TiO_2_ thicknesses. The transmittance gradually decreases as the TiO_2_ thickness increases over the entire wavelengths (λ). However, at λ ~ 550 nm, decrease in the transmittance of 10 and 20 nm-thick TiO_2_ coated TCE is less than 5 %. Interestingly, the sheet resistance maintains to be 80 Ω/sq until the TiO_2_ thickness reaches to 20 nm, after then, it increases rapidly. We tracked the sheet resistance of the passivated TCE during 2 months as shown in Fig. 4c. The 20 nm-thick TiO_2_ shell perfectly passivated the Ag-NWs from oxidation, evidenced by the constant sheet resistance, while the 10 nm-thick shell resulted in 50 % of increase in sheet resistance within 2 months. Moreover, the 20 nm-thick TiO_2_-coated Ag-NW TCE exhibits an excellent bending durability. Figure 4d shows the sheet resistances, as a function of bending cycles, of our flexible Ag-NW TCE (20 nm TiO_2_) and commercial flexible ITO film. At initial the sheet resistance of the ITO film is much lower than that of our TCE. However, the sheet resistance of the ITO film significantly increases to >300 Ω/sq after 30 cycles of bending, while that of our TCE keeps identical sheet resistance after the 30 cycles of bending test. Lastly, we tested the flexibility of a real solar cell device made with our Ag-NW TCE (20 nm TiO_2_). A ssDSSC was fabricated using our TCE (Fig. 4e), with a low-temperature processed mesoporous TiO_2_ thick film as an electron transport layer, Z907 dye as a sensitizer, and (2,20,7,70-tetrakis (*N*,*N*-di-*p*-methoxyphenylamine)-9-90-spiro-bifluorene)(spiro-OMeTAD) as a hole transport layer (details in experimental part). In spite of the excellent flexibility of our Ag-NW TCE (20 nm TiO_2_), the ssDSSC based on it shows poor bending durability (Fig. 4f). The as-fabricated ssDSSC exhibits 0.37 mA/cm^2^ of short circuit current density (*J*_sc_), 0.541 V of open circuit voltage (*V*_oc_), 0.40 of fill factor, and 0.08 % of power conversion efficiency (PCE), and all the performances degraded to 0.30 mA/cm^2^ of *J*_sc_, 0.529 V of *V*_oc_, 0.32 of fill factor, and 0.05 % of PCE after 100 cycles of bending. Because our TCE itself has good bending durability as shown in Fig. 4d, we attribute the poor bending durability of the ssDSSC to a weak bending durability of the mesoporous TiO_2_ layer which is a ceramic that might have a brittle nature.

## Conclusions

We fabricated a highly stable and flexible Ag-NW-based TCE by using TiO_2_ nanoshell which effectively passivates Ag from oxidation in ambient air. The Ag-NWs were synthesized via polyol method, and bar-coated on a flexible PET substrate, followed by laminating and ALD of TiO_2_ layer. The 20 nm-thick TiO_2_ barrier perfectly passivates the oxidation of Ag-NWs, showing less than 0.01 % of sheet resistance change during 2 months of aging in ambient air, whereas the bare Ag-NW TCE shows >150 % of sheet resistance increase under the identical condition. The 20 nm-thick TiO_2_-coated Ag-NW TCE exhibits a high transmittance >75 % at λ ~ 550 nm, a low sheet resistance ~80 Ω/sq, and an excellent bending durability, i.e. constant sheet resistance after 30 cycles of bending test (under 30 mm of bending radius). Furthermore, we demonstrate the potential of our passivated Ag-NW TCE for real application to flexible devices such as ssDSSC.

## Experimental details

### Fabrication of Ag-NW TCE

Ag-NWs were synthesized as follows. 0.5 mL PtCl_2_ solution (1.5 × 10^−4^ M) which was added into 5 mL ethylene glycol solvent, with stirring at 170 °C. After 4 min of stirring, 2.5 mL AgNO_3_ solution (0.12 M) and 5 mL polyvinylpyrrolidone (PVP) solution (0.36 M) was added dropwise with maintaining the reaction temperature fixed at 170 °C. After slow cooling this mixture, the residual PVP was eliminated by centrifuging (6000 rpm and 30 min duration). Then this sediment was redispersed in methanol. The average dimension of the synthesized Ag-NWs was 10 μm in length and 80 nm in diameter.

The Ag-NW solution was coated onto polyethylene terephthalate (PET) films uniformly using a Teflon rod and repeated this process 4–6 times after the Ag-NW films were dried under the IR lamp. Then, the Ag-NW films were laminated under pressure of 200 kg/cm^2^ at 120 °C.

To retard the oxidation of Ag-NW, TiO_2_ barrier was uniformly deposited by an atomic layer deposition (ALD) system. Titanium (IV) isopropoxide (TTIP, UPChem) was used as the Ti precursor and H_2_O was employed as the oxygen source. High purity Ar was used a purge gas and to carry the TTIP. Each cycle of deposition was comprised of 10 s of pre-purging, 3 s of TTIP source injection, and 1 s of H_2_O flow.

### Fabrication of flexible ssDSSC

TiO_2_ paste (Dyesol 18 NR-T) was coated on the fabricated Ag-NW TCE by doctor blade and dried at 80 °C in ambient air, followed by atmospheric pressure plasma treatment [16] for 30 min. The fabricated film was soaked in Z907 dye at 50 °C for 2 h and rinsed with ethanol. A hole transport layer (80 mg of 2,2′,7,7′-tetrakis(*N*,*N*-di-*p*-methoxyphenyl-amine)-9,9′-spirobifluorene (spiro-MeOTAD), 8.4 μL of 4-*tert*-butylpyridine, and 51.6 μL of bis (trifluoro-methane) sulfonimide lithium salt (Li-TFSI) solution (154 mg/mL in acetonitrile), the whole mixture was dissolved in 1 mL chlorobenzene) was formed after spin coating at 2000 rpm for 45 s. Au electrode was deposited by thermal evaporation under 10^−6^ bar with a shadow mask. J-V curves of the fabricated ssDSSC were measured using a potentiostat under the simulated sun light (AM 1.5, 100 mW/cm^2^).
